# Supplementary material for: Increasing altruistic and cooperative behaviour with simple moral nudges
Source: Sci Rep. 2019 Aug 15;9:11880. doi: 10.1038/s41598-019-48094-4 (PMC6695418; doi:10.1038/s41598-019-48094-4)
Supplement: Supplementary file 1 — Detailed statistical analysis and Experimental instructions [file 41598_2019_48094_MOESM1_ESM.docx]

**Supplementary Material**

for

**Increasing altruistic and cooperative behaviour with simple moral nudges**

Valerio Capraro^1^, Glorianna Jagfeld^2^, Rana Klein^3^, Mathijs Mul^3^ & Iris van de Pol^3^

^1^Middlesex University of London. ^2^Universität Stuttgart. ^3^Universiteit van Amsterdam.

This Supplementary Material is divided in two parts. In the first part, we report details of the statistical analysis. In the second part, we report full experimental instructions.

**Part SM1. Statistical analysis**

**Table SM1. Linear regressions predicting Dictator Game altruism in Study 1.** Participants were randomly assigned to one of three conditions: DG, DGdescriptive, and DGpersonal. We denote “nudged” the union between DGdescriptive and DGpersonal. Column (1) shows that being nudged has a significant positive effect on DG altruism. Column (2) shows that this effect is robust after controlling for sex, age, and education. Column (3) shows that the “social nudge” has a significant (at 10%) positive effect on DG altruism. Column (4) demonstrates that this effect is robust and actually is strengthened after controlling for sex, age, and education. Columns (5) and (6) show that the “personal nudge” has a significant positive effect on DG donations.

|  | **Dictator Game altruism** | | | | | |
| --- | --- | --- | --- | --- | --- | --- |
|  | **(1)** | **(2)** | **(3)** | **(4)** | **(5)** | **(6)** |
| **nudged** | 0.094***  (0.033) | 0.104***  (0.033) |  |  |  |  |
| **DGdescriptive** |  |  | 0.074*  (0.039) | 0.086**  (0.039) |  |  |
| **DGpersonal** |  |  |  |  | 0.114***  (0.037) | 0.122***  (0.037) |
| **sex** |  | 0.064**  (0.032) |  | 0.019  (0.041) |  | 0.060  (0.038) |
| **age** |  | 0.002  (0.002) |  | 0.003  (0.002) |  | 0.002  (0.002) |
| **education** |  | -0.014  (0.012) |  | -0.017  (0.015) |  | -0.021  (0.014) |
| **constant** | 0.212***  (0.027) | 0.103  (0.081) | 0.212***  (0.028) | 0.152  (0.099) | 0.212***  (0.027) | 0.149  (0.094) |
| **obs** | 282 | 282 | 188 | 188 | 184 | 184 |
| **r-squared** | 0.028 | 0.056 | 0.019 | 0.041 | 0.048 | 0.078 |

Note: Linear regression, robust standard error in parentheses. *: significant at 10%, **: significant at 5%, ***: significant at 1%.

**Table SM2. Linear regression predicting Prisoner’s Dilemma cooperation in Study 2.** Participants were randomly assigned to one of three conditions: PD, PDdescriptive, and PDpersonal. We denote “nudged” the union between PDdescriptive and PDpersonal. Column (1) shows that being nudged has a significant positive effect on PD cooperation. Column (2) shows that this effect is robust after controlling for sex, age, and education. Columns (3) and (4) show that the “social nudge” has a significant positive effect on PD cooperation, and that this effect is robust after controlling for sex, age, and education. Columns (5) and (6) show that the “personal nudge” does not have a significant positive effect on PD cooperation (the effect, however, is close to marginally significant: both p’s<0.13. Moreover, comparing PDdescriptive with PDpersonal, we find no significant difference: p=0.327).

|  | **Prisoner’s Dilemma cooperation** | | | | | |
| --- | --- | --- | --- | --- | --- | --- |
|  | **(1)** | **(2)** | **(3)** | **(4)** | **(5)** | **(6)** |
| **nudged** | 0.151**  (0.066) | 0.138**  (0.066) |  |  |  |  |
| **PDdescriptive** |  |  | 0.188**  (0.075) | 0.164**  (0.077) |  |  |
| **PDpersonal** |  |  |  |  | 0.114  (0.075) | 0.115  (0.072) |
| **sex** |  | 0.126**  (0.061) |  | 0.049  (0.075) |  | 0.219***  (0.074) |
| **age** |  | 0.005*  (0.003) |  | 0.003  (0.004) |  | 0.008**  (0.004) |
| **education** |  | 0.016  (0.025) |  | -0.038  (0.032) |  | -0.011  (0.028) |
| **constant** | 0.329***  (0.054) | -0.096  (0.168) | 0.330***  (0.054) | 0.011  (0.123) | 0.330***  (0.054) | -0.197  (0.192) |
| **obs** | 257 | 257 | 169 | 169 | 170 | 170 |
| **r-squared** | 0.020 | 0.053 | 0.036 | 0.041 | 0.014 | 0.103 |

Note: Linear regression, with robust standard error in parentheses. *: significant at 10%, **: significant at 5%, ***: significant at 1%.

**Table SM3. Linear regression predicting second-stage Dictator Game altruism in Study 3.** Participants were randomly assigned to one of two two-stage games: DG-DG2, DGpersonal-DG2. We regress second-stage behaviour as a function of whether subjects, in stage 1, participated in the DGpersonal condition or in the DG condition. Column (1) shows that who participated in the DGpersonal condition gave, in stage 2, more than those who participated in the DG condition. Column (2) shows that this effect is robust after controlling for sex, age, and education.

|  | **Stage 2 Dictator Game altruism** | |
| --- | --- | --- |
|  | **(1)** | **(2)** |
| **DGpersonal** | 0.075**  (0.036) | 0.086**  (0.036) |
| **sex** |  | 0.050  (0.035) |
| **age** |  | 0.004**  (0.001) |
| **education** |  | 0.012  (0.015) |
| **constant** | 0.180***  (0.024) | -0.086  (0.109) |
| **obs** | 172 | 172 |
| **r-squared** | 0.025 | 0.073 |

Note: Linear regression, with robust standard error in parentheses. *: significant at 10%, **: significant at 5%, ***: significant at 1%.

**Table SM4. List of all direct effects.** Summary of all first stage manipulations we have conducted in Studies 1-4. For each manipulation, the treatment is compared with the baseline.

|  | **OLS coefficients** | |
| --- | --- | --- |
|  | **(1)** | **(2)** |
| **DGpersonal (study 1)** | 0.114***  (0.037) | 0.122***  (0.037) |
| **DGpersonal (study 3)** | 0.059  (0.037) | 0.068*  (0.037) |
| **DGpersonal (study 4)** | 0.091**  (0.040) | 0.097**  (0.041) |
| **DGdescriptive (study 1)** | 0.074*  (0.039) | 0.086**  (0.039) |
| **DGdescriptive (study 4)** | 0.061  (0.040) | 0.063  (0.040) |
| **PDpersonal (study 2)** | 0.114  (0.075) | 0.115  (0.072) |
| **PDpersonal (study 4)** | 0.075  (0.079) | 0.061  (0.080) |
| **PDdescriptive (study 2)** | 0.188**  (0.075) | 0.164**  (0.077) |
| **PDdescriptive (study 4)** | 0.146*  (0.078) | 0.135*  (0.078) |
| **overall effect on DG (meta-analysis)** | 0.080*** | 0.088*** |
| **overall effect on PD (meta-analysis)** | 0.132*** | 0.120*** |
| **control on demographics** | no | yes |

Note: Linear regression, with robust standard error in parentheses. *: significant at 10%, **: significant at 5%, ***: significant at 1%.

**Table SM5. List of all spillover effects.** Summary of all spillover effects in Study 4. Since spillover effects in Stage 2 depend on Stage 1 direct effects, in order to compute the coefficients of the spillover effect we use second-stage linear regression, which allows us to express second-stage effects as a proportion of first stage effects. To use second-stage linear regression, we need to re-normalize DG donations by multiplying them by 2 (because PD cooperation goes from 0 to 1, while DG altruism goes from 0 to 0.5). For each manipulation, the treatment is compared with the baseline. Spillover effects are all in the same direction, although none of them is statistically significant when taken singularly. However, when meta-analyzing them together, we find a significant effect which is also big in size.

|  | **OLS coefficients** | |
| --- | --- | --- |
|  | **(1)** | **(2)** |
| **DGpersonal-PD** | 0.694  (0.432) | 0.653  (0.415) |
| **DGdescriptive-PD** | 0.658  (0.648) | 0.647  (0.634) |
| **PDpersonal-DG** | 0.166  (1.191) | 0.451  (1.470) |
| **PDdescriptive-DG** | 0.553  (0.587) | 0.604  (0.641) |
| **overall effect (meta-analysis)** | 0.618** | 0.633** |
| **control on demographics** | no | yes |

Note: Second-stage linear regression with robust standard error in parentheses. *: significant at 10%, **: significant at 5%, ***: significant at 1%.


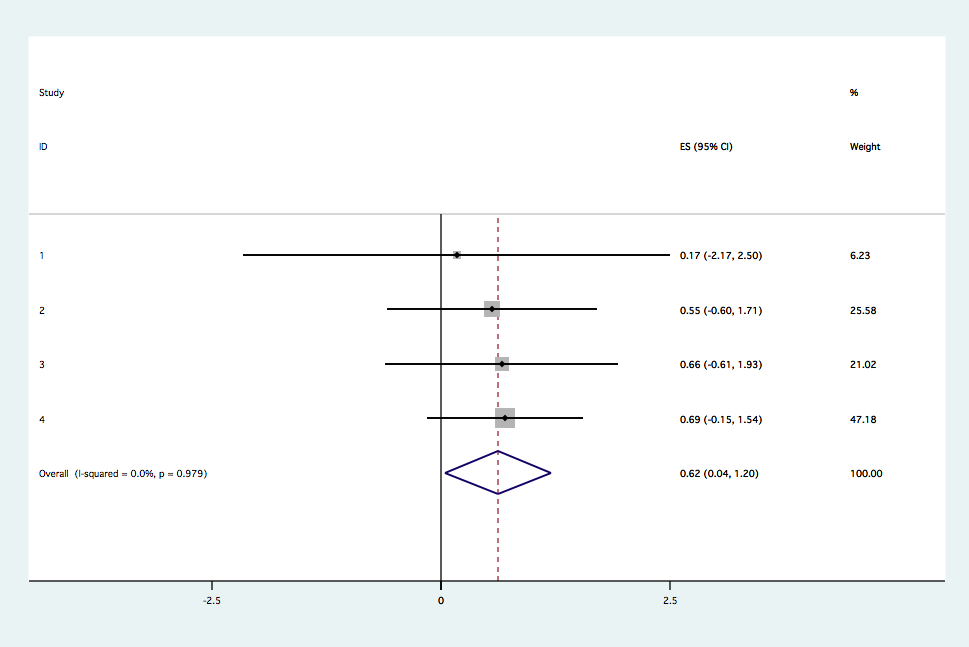


**Figure SM1: Forest plot of the meta-analysis of Study 4***. Study ID = 1 compares average cooperation in PD played after DG personal with average cooperation in PD played after DG neutral. Study ID = 2 compares average cooperation in PD played after DG social with average cooperation in PD played after DG neutral. Study ID = 3 compares average altruism in DG played after PD social with average altruism in DG played after PD neutral; finally, Study ID = 4 compares average altruism in DG played after PD personal with average altruism in DG played after PD neutral. All the effects are in the same direction, meaning that in all four cases, the average prosociality in games played in Stage 2 after a moral nudge is implemented in Stage 1 is numerically higher than average prosociality in games played in Stage 2 after no moral nudge is implemented in Stage 1. None of these effects is statistically significant when taken singularly (DG after PD vs DG after PDpersonal: p = 0.889; DG after PD vs DG after PDdescriptive: p = 0.348; PD after DG vs PD after DGpersonal: p = 0.110; PD after DG vs PD after DGdescriptive: p = 0.309). However, meta-analysis shows that there is a significant overall effect (overall effect size = 0.633, 95% CI [0.047,1.219], Z = 2.12, p = 0.034), that is also big in size: about 63% of the original effect of moral nudges spill across contexts.*

**Part SM2. Experimental instructions**

**Study 1**

*DG instructions and comprehension questions (common to all treatments)*

Please read these instructions carefully. You may earn a considerable bonus, depending on the decision you make.

You have been paired with another participant. The amount of money you can earn depends only on your choice. You are given 20c and the other participant is given nothing. You have to decide how much, if any, to transfer to the other participant.

The other participant is REAL, and has no choice but to accept the amount of money you decide to transfer.

No deception is used. You will really get the amount of money you decide to keep.

Here are some questions to ascertain that you understand the rules. Remember that you have to answer all of these questions correctly in order to get the completion code. If you fail any of them, the survey will automatically end and you will not have a chance to earn a bonus.

What is the transfer by YOU that maximizes YOUR bonus? (Available answers: 0c/2c/…/20c)

What is the transfer by YOU that maximizes the OTHER PARTICIPANT's bonus? (Available answers: 0c/2c/…/20c)

(Here there was a display logic, such that participants were automatically eliminated if they failed one of the previous comprehension questions. In case they were not eliminated, the survey proceeded to the next screen)

Congratulations, you have answered both comprehension questions correctly!

*DG neutral*

It is now time to make your choice.

What amount will you transfer to the other person? (Available answers: 0c/2c/…/20c)

*DG personal*

What do you personally think is the morally right thing to do in this situation?

Transfer:

(Available answers: 0c/2c/…/20c)

It is now time to make your choice.

What amount will you transfer to the other person? (Available answers: 0c/2c/…/20c)

*DG social*

What do you think your society considers to be the morally right thing to do in this situation?

Transfer:

(Available answers: 0c/2c/…/20c)

It is now time to make your choice.

What amount will you transfer to the other person? (Available answers: 0c/2c/…/20c)

**Study 2**

*PD instructions and comprehension questions (common to all treatments)*

Please read these instructions carefully. You may earn a considerable bonus, depending on the decisions you and another participant make.

You have been paired with another anonymous participant. You are both given 10c and each of you must decide whether to transfer the 10c or not. If a participant transfers their 10c, this amount will be doubled, and given to the other participant.

So:

- If you both decide to transfer the 10c, you end this part of the survey with a bonus of 20c.
- If the other participant transfers the 10c and you do not, you end this part of the survey with a bonus of 30c.
- If you transfer the 10c and the other participant does not, you end this part of the survey with a bonus of 0c.
- If neither of you transfer the 10c, then you end this part of the survey with a bonus of 10c.

The other participant is REAL, and you and the other participant are really going to get a bonus depending on the decisions that you and the other participant will make.

Here are some questions to ascertain that you understand the rules. Remember that you have to answer all of these questions correctly in order to get the completion code. If you fail any of them, the survey will automatically end and you will not have a chance to earn a bonus.

What choice should YOU make to maximise YOUR gain? (available answers: transfer the 10c/don’t transfer the 10c)

What choice should YOU make to maximise the OTHER PARTICIPANT's gain? (available answers: transfer the 10c/don’t transfer the 10c)

What choice should the OTHER PARTICIPANT make to maximise THEIR gain? (available answers: transfer the 10c/don’t transfer the 10c)

What choice should the OTHER PARTICIPANT make to maximise YOUR gain? (available answers: transfer the 10c/don’t transfer the 10c)

(Here there was a display logic, such that participants were automatically eliminated if they failed one of the previous comprehension questions. In case they were not eliminated, the survey proceeded to the next screen)

Congratulations, you have answered both comprehension questions correctly!

*PD neutral*

It is now time to make your choice. (available answers: transfer the 10c/don’t transfer the 10c)

*PD personal*

What do you personally think is the morally right thing to do in this situation? (available answers: transfer the 10c/don’t transfer the 10c)

It is now time to make your choice. (available answers: transfer the 10c/don’t transfer the 10c)

*PD social*

What do you think your society considers to be the morally right think to do in this situation? (available answers: transfer the 10c/don’t transfer the 10c)

It is now time to make your choice. (available answers: transfer the 10c/don’t transfer the 10c)

**Study 3**

The DG and DGpersonal conditions were identical to those of Study 1.

*DG2 condition (common to all participants)*

This is the second part of the HIT.

Please read these instructions carefully. You may earn a considerable bonus, depending on the decision you make.

You have been paired with another participant, different from the one you were paired with in the previous part of the HIT. The amount of money you can earn depends only on your choice. You are given 40c and the other participant is given nothing. You have to decide how much, if any, to transfer to the other participant.

The other participant is REAL, and has no choice but to accept the amount of money you decide to transfer.

No deception is used. You will really get the amount of money you decide to keep.

What amount will you transfer to the other person? (Available answers: 0c/4c/8c/…/40c)

**Study 4**

The PD, PDdescriptive, PDpersonal, DG, DGdescriptive, and DGpersonal conditions are identical to those of Study 1 and Study 2.

**Study 5, Session 1**

*Welcome screen (common to all subjects)*

Welcome to this HIT.

This HIT consists of a set of questions. Answering these questions will take about five minutes.

For the participation in this HIT, you will earn 50c.

With this in mind, do you wish to continue?

*Survey (common to all subjects whose goal is to create a sense of endowing the 50c)*

Gender: (available answers: male/female)

Age: (open answer)

Highest level of education completed: (available answers: less than a high school degree/high school diploma/vocational training/attended college/bachelor’s degree/graduate degree/unknown)

Please choose the category that describes the total amount of income you earned in 2015. Consider all forms of income, including salaries, tips, interest and dividend payments, scholarship support, student loans, parental support, social security, alimony, and child support, and others. ($5,000-$10,000/$10,001-$15,000/$15,001-$25,000/$25,001-$35,000/$35,001-$50,000/$50,001-$65,000/$65,001-$80,000/$80,001-$100,000/over $100,000)

How do you see yourself: are you generally a person who is fully prepared to take risks or do you try to avoid taking risks? (11-point likert-scale from 0=not at all willing to take risks to 10=very willing to take risks)

To what extent do you feel you can trust other people that you interact with in your daily life? (7-point likert scale from 1=very little t0 7=very much)

I would rather do something that requires little thought than something that is sure to challenge my thinking abilities. (7-point likert scale from 1=very untrue to 7=very true)

I trust my initial feelings about people. (7-point likert scale from 1=very untrue to 7=very true)

Which US political party do you identify with more strongly? (7-point likert scale from 1=strongly Republican to 4=neutral to 7=strongly Democratic)

How strongly do you believe in the existence of a God or Gods? (7-point likert scale from 1=very little to 7=very much)

Politically, how conservative are you in terms of social issues? (6-point likert scale from 1=very liberal to 6=very conservative)

Politically, how conservative are you in terms of fiscal issues? (6-point likert scale from 1=very liberal to 6=very conservative)

*Neutral*

Thanks for answering our questions

*Nudged*

If you see a stranger in need, what do you think is the morally right thing to do? (available answers: help/don’t help)

*Donation to Emergency*

We are collecting donations to send to Emergency. Emergency is a humanitarian NGO that provides emergency medical treatment to civilians victims of war. For more information, visit: www.emergencyusa.org

We would like to ask you if you would be willing to renounce to your 50c participation fee and donate it to Emergency. If you do so, we will not provide you a completion code so as you will not be allowed to submit the HIT. This means that your submission will NOT be rejected. Simply, your HIT will not be submitted.

(available answers: No, I do not want to donate my 50c participation fee to Emergency/Yes, I want to donate my 50c participation fee to Emergency)

*Donation to Give for France*

You have probably heard about the terroristic attack in Nice last night, which killed at least 84 people, including several children.

We are collecting donations to send to Give For France. Give For France is a campaign to help victims of the Nice attack. For more information, visit: http://www.giveforfrance.org/en

We would like to ask you if you would be willing to renounce to your 50c participation fee and donate it to Give For France. If you do so, we will not provide you a completion code so as you will not be allowed to submit the HIT. This means that your submission will NOT be rejected. Simply, your HIT will not be submitted.

(available answers: No, I do not want to donate my 50c participation fee to Give for France/Yes, I want to donate my 50c participation fee to Give for France)

**Study 5, Session 2**

The survey of Session 2 is identical to that of Session 1. The remaining part of the survey is slightly different.

*Neutral*

Thanks for answering our questions

*Nudged*

You are given 10c and an anonymous stranger is given nothing. You have the chance to give part of your money to the stranger.

The stranger has no choice: she or he has no chance to reciprocate your action.

What amount do you think it would be morally right to give to the stranger? (available answers: 0c/1c/2c/…/10c)

*Donation to Emergency*

We are collecting donations to send to Emergency. Emergency is a humanitarian NGO that provides emergency medical treatment to civilians victims of war. For more information, visit: www.emergencyusa.org

We would like to ask you if you would be willing to donate part of your 50c participation fee to Emergency.

If you decide not to donate money, we will provide you a completion code that you can use to submit this HIT to AMT and claim for the whole 50c.

If you decide to donate some money, we will not provide you a completion code for this survey. In order to pay you the amount that you decided to keep for yourself, we will redirect you to another survey, in which you will get this amount paid as a bonus.

How many cents would you like to donate to Emergency?

Please insert only a number between 0 and 50.

(here there is a textbox in which participants could type their donation)

*Donation to Give for France*

You have probably heard about the terroristic attack in Nice last night, which killed at least 84 people, including several children.

We are collecting donations to send to Give For France. Give For France is a campaign to help victims of the Nice attack. For more information, visit: http://www.giveforfrance.org/en

We would like to ask you if you would be willing to donate part of your 50c participation fee to Give For France.

If you decide not to donate money, we will provide you a completion code that you can use to submit to AMT and claim for the whole 50c.

If you decide to donate some money, we will not provide you a completion code for this survey. In order to pay you the amount that you decided to keep for yourself, we will redirect you to another survey, in which you will get this amount paid as a bonus.

How many cents would you like to donate to Give For France?

Please insert only a number between 0 and 50.

(here there is a textbox in which participants could type their donation)

**Study 5, Session 3**

The survey is identical to that of the previous sessions. The “donation to Emergency” and “donation to Give for France” screens are identical to those of Session 2. The “neutral” and “nudged” screen are slightly different.

*Neutral*

Consider the expression 10 - x, for x that goes from 0 to 10.

Thus:

- if x = 0, then 10 - x = 0,
- if x = 1, then 10 - x = 9,
- …
- if x = 9, then 10 - x = 1,
- if x = 10, then 10 - x = 0.

We will now ask you two simple comprehension questions to make sure that you understand the situation.

What number x, between 0 and 10, MAXIMIZES the equation 10 - x? (available answers: 0/1/2/…/10)

What number x, between 0 and 10, MINIMIZES the equation 10 - x? (available answers: 0/1/2/…/10)

*Nudged*

You are given 10c and an anonymous stranger is given nothing. You have the chance to give part of your money to the stranger.

The stranger has no choice: she or he has no chance to reciprocate your action.

We will now ask you some questions to make sure that you understand the situation.

What amount should you give to the stranger in order to maximize your payoff?

What amount should you give to the stranger in order to maximize their payoff?

The comprehension questions are over.

It's time to make your real choice.

What do you think is the morally right thing to do? (available answers: Give 0c/Give 1c/Give 2c/…/Give 10c)
